# Influence of Household Rat Infestation on *Leptospira* Transmission in the Urban Slum Environment

**DOI:** 10.1371/journal.pntd.0003338

**Published:** 2014-12-04

**Authors:** Federico Costa, Guilherme S. Ribeiro, Ridalva D. M. Felzemburgh, Norlan Santos, Renato Barbosa Reis, Andreia C. Santos, Deborah Bittencourt Mothe Fraga, Wildo N. Araujo, Carlos Santana, James E. Childs, Mitermayer G. Reis, Albert I. Ko

**Affiliations:** 1 Centro de Pesquisas Gonçalo Moniz, Fundação Oswaldo Cruz, Ministério da Saúde, Salvador, Bahia, Brazil; 2 Department of Epidemiology of Microbial Diseases, School of Public Health, Yale University, New Haven, Connecticut, United States of America; 3 Instituto de Saúde Coletiva, Universidade Federal da Bahia, Bahia, Brazil; 4 Federal University of Bahia, Salvador, Bahia, Brazil; 5 University of Brasília, Ceilândia, Distrito Federal, Brazil; 6 Zoonosis Control Center, Brazil Ministry of Health, Salvador, Bahia, Brazil; University of Tennessee, United States of America

## Abstract

**Background:**

The Norway rat (*Rattus norvegicus*) is the principal reservoir for leptospirosis in many urban settings. Few studies have identified markers for rat infestation in slum environments while none have evaluated the association between household rat infestation and *Leptospira* infection in humans or the use of infestation markers as a predictive model to stratify risk for leptospirosis.

**Methodology/Principal Findings:**

We enrolled a cohort of 2,003 urban slum residents from Salvador, Brazil in 2004, and followed the cohort during four annual serosurveys to identify serologic evidence for *Leptospira* infection. In 2007, we performed rodent infestation and environmental surveys of 80 case households, in which resided at least one individual with *Leptospira* infection, and 109 control households. In the case-control study, signs of rodent infestation were identified in 78% and 42% of the households, respectively. Regression modeling identified the presence of *R. norvegicus* feces (OR, 4.95; 95% CI, 2.13–11.47), rodent burrows (2.80; 1.06–7.36), access to water (2.79; 1.28–6.09), and un-plastered walls (2.71; 1.21–6.04) as independent risk factors associated with *Leptospira* infection in a household. We developed a predictive model for infection, based on assigning scores to each of the rodent infestation risk factors. Receiver operating characteristic curve analysis found that the prediction score produced a good/excellent fit based on an area under the curve of 0.78 (0.71–0.84).

**Conclusions/Significance:**

Our study found that a high proportion of slum households were infested with *R. norvegicus* and that rat infestation was significantly associated with the risk of *Leptospira* infection, indicating that high level transmission occurs among slum households. We developed an easily applicable prediction score based on rat infestation markers, which identified households with highest infection risk. The use of the prediction score in community-based screening may therefore be an effective risk stratification strategy for targeting control measures in slum settings of high leptospirosis transmission.

## Author Summary

The Norway rat is an important reservoir for urban leptospirosis, a life-threatening zoonotic disease. In urban settings, leptospirosis transmission occurs primarily in the peri-domiciliary environment of the slums. Rodent control is one of the most frequent strategies to prevent leptospirosis, but the identification of domiciles at higher risk of transmission is challenging. We compared households where an individual with evidence of recent leptospirosis infection resided and households where none of the residents had evidence for infection. Houses with evidence of leptospirosis transmission had higher levels of rodent infestation and environmental conditions related to rodents. We propose a new methodology to easily characterize slum households, based on environmental characteristics, at different levels of risk for leptospirosis transmission. The findings of this study indicate that evaluation for rodent infestation intensity and environmental features may be a feasible strategy for targeting augmented control measures for leptospirosis.

## Introduction

In developing countries, leptospirosis is an emerging health problem affecting urban slum communities [1]–[4]. Annual epidemics of the disease typically occur during periods of seasonal rainfall [1], [5]–[8]. Lack of sanitation infrastructure such as open sewage systems and poor refuse collection services provide conditions for proliferation of rats, which are the main reservoir for leptospirosis in urban settings [1], [9]–[11]

Pathogenic *Leptospira* infection produces a broad spectrum of clinical manifestations with case fatality exceeding 10% and 50% for Weil's disease and severe pulmonary hemorrhage syndrome, respectively [2], [12], [13]. Currently, there are no effective interventions which can be easily implemented in slum communities to prevent leptospirosis transmission. Rat-control programs are commonly implemented as a control measure for leptospirosis in many cities, such as those in Brazil, but their effectiveness is questionable and has not been systematically explored.

In this setting, two rat species, the Norway rat (*Rattus norvegicus*) and black rat (*Rattus rattus*), are the main reservoirs for this bacterium and contaminate environments via urinary shedding, providing conditions for transmission to humans [2]. Prior studies in urban areas have shown that *Leptospira* carriage ranges between 7–82% for *R. norvegicus* [14], [15] and between 7–34% for *R. rattus* [16], [17]. However, the Norway rat is far more common within the urban slum environments: nearly 100% of rats trapped in the city of Salvador, Brazil comprised of this species [14], [15], [18]. *Leptospira* strains isolated from Norway rats were genotypically identical with strains obtained from human patients based on PCR-based typing methods [19]. Additionally, epidemiological studies have found that peri-domiciliary resident reporting of rat sightings and living in proximity to open sewers placed residents at increased risk for leptospiral transmission in slum areas [5], [10]. These findings support the role of urban peri-domestic transmission due to contact with water contaminated with rat urine.

Rodent control programs based on environmental application of a chemical rodenticide [20] as an strategy to reduce the incidence of leptospirosis are costly and their effectiveness has not been evaluated [20], [21]. Programs implemented in Brazil [20] are based on the Centers for Disease Control and Prevention (CDC) approach for pest management [22] which includes an environmental form to assess rodent infestation levels and infrastructural deficiencies in peridomestic areas. Nevertheless, the CDC survey form has not been validated for application in slum areas of developing countries. Furthermore, no studies have systematically examined whether indicators of rodent infestation assessed during rodent surveys can be used to predict leptospirosis risk and, therefore, guide targeted interventions specifically implemented among high risk households. Herein, by using a community-based cohort study aimed to evaluate *Leptospira* infection, we describe an environmental and rodent survey instrument, adapted from CDC guidelines, for use in a tropical slum area. In addition, we developed and evaluated the accuracy of a scoring system to predict *Leptospira* transmission using data easily produced by this instrument.

## Methods

### Study site

**Figure 1:**
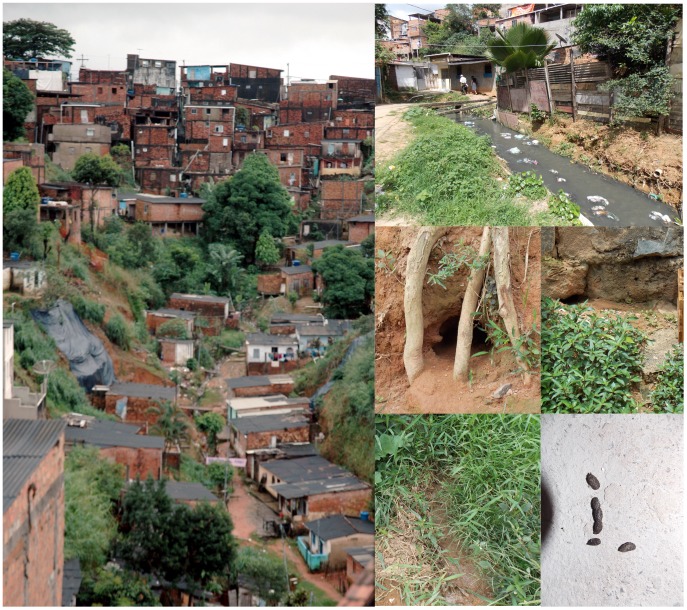
Environmental variables related to source of food, water, harborage and access for rodents and rodent active signs in the study area. (A and B) Photographs of the typical environment at the community study site, which shows a valley in which households are situated and the proximity of households to open sewers, exposed garbage and bushes or shrubbery. (C and D) Rodent burrows. (D and E) Rodent runs. (F) *Rattus norvegicus* fecal droppings.

This study was conducted in Pau da Lima, a slum community situated in the periphery of Salvador, a city of 2.7 million inhabitants [23] in Northeast Brazil. This site was selected for epidemiological studies on leptospirosis based on the high annual incidence of severe leptospirosis (35.4 cases per 100,000 pop.) identified in this community by active surveillance during1996 to 2002. The study site is a four valley area of 0.46 km^2^, characterized by the absence of basic sanitation and high levels of rat infestation [10] (Figure 1A–B). In 2003, we conducted a census in the area and identified 14,122 residents living at 3,689 households. The socioeconomic profile in this site was similar to other slum populations in Brazil: subjects were squatters (85%) who did not complete primary school (77%) and had a median household per capita income of $1.30 per day.

### Study design and cohort investigation

We performed a case-control study of households in the Pau da Lima community in order to evaluate the association between household environmental characteristics and rodent infestation on household-level *Leptospira* infection risk. Households were selected among those which participated in a prospective community-based cohort study designed to characterize the burden of *Leptospira* infection [11]. This cohort investigation, performed between 2003 and 2007, comprised in part of four annual serosurveys of 2,003 cohort subjects who were greater than five years of age and resided in 684 (18.5% of 3,689) randomly-selected households at the study site [11].

The microscopic agglutination test (MAT) was performed on serum samples from the baseline survey (initiated in 2003 and completed in 2004) and follow-up surveys (2004/2005, 2005/2006 and 2006/2007) to identify subjects with serologic evidence of a recent *Leptospira* infection. As previously described [10], [11], MAT evaluations were performed with a panel of five reference strains and two clinical isolates [1], which included *L. interrogans serovars* Autumnalis, Canicola and Copenhageni; *L. borgspetersenii* serovar Ballum, and *L. kirschneri* serovar Grippotyphosa. All sera were screened at dilutions of 1∶25, 1∶50 and 1∶100. Positive samples at a dilution of 1∶100 were titrated to determine the endpoint agglutination titer. A recent leptospiral infection was defined as seroconversion during which the MAT titer increased from negative at the baseline survey to a titer ≥1∶50 during the follow-up survey or as a four-fold rise in MAT titer in a participant with a titer of ≥1∶25 during the baseline survey [11].

All cohort members provided written informed consent before enrollment. Minors (<18 years of age) provided assent to participate, in addition to informed consent from their legal guardian. Ethical clearance for this study was provided by the Ethical Committee in Research of the Oswaldo Cruz Foundation and IRB committees of Weill Medical College of Cornell University and the Yale School of Public Health.

### Selection of case and control households

A case household was defined by occurrence of at least one *Leptospira* infection event among subject residents of the household during the 3 years of follow-up. Identification of case households were performed after completion of the three years of cohort follow-up. A random number table was used to select control households (1∶1 case∶control ratio) among those at the Pau da Lima site which fulfilled the following criteria: a) absence of *Leptospira* infection event among cohort subjects who were members of the household, b) the presence of at least one household member who participated for the duration of the cohort study, and c) households situated ≥30 m from the nearest case household. The criterion of 30 m was selected to minimize the possibility of overlapping rat infestations as the typical home range of *R. norvegicus* varies between 30–50 m in urban areas [24], [25]. Control households were selected after completion of the three years of cohort follow-up, just after identification of case households.

### Rodent infestation survey

Environmental surveys of case and control households were conducted by a team of rodent control specialists from the municipal Zoonosis Control Center (ZCC) in Salvador. Study houses and peri-domestic areas (10 m around each household) were surveyed between October and November of 2007. The survey team used a modified exterior inspection form, adapted from the CDC manual (Table S1) [22]. The form included the following six groups of variables: a) 7 variables on premise type; b) 5 variables on food sources for rodents; c) 3 variables on water sources for rodents; d) 11 variables on harborage for rodents; e) 5 variables on entry/access for rodents and f) 6 variables on signs of rodent infestation (Table S1, available in English and Portuguese; Figure 1C–F). Team performing the surveys was blinded regarding household case status.

### Household data collection

In addition to the environmental survey, we administered a standardized questionnaire to the head-of-the-household, which included 4 questions on demographics and 4 on ownership and number of domestic animals. Because of the time lag between occurrence of *Leptospira* infection and the household rodent survey, we asked the head-of-household if domicile or peridomicile structure changes had occurred (i.e. rebuilding or expansion), if nearby open sewers were closed/created and if refuse deposits were removed/created since the year of *Leptospira* infection. Year of infection for case households was available to the field team in order to perform the questionnaire, and to maintain blinding, we generated random years to serve as sham infection dates for surveys of control households.

During September 2007, an environmental inspection was performed in the entire study area. Location of study households, in addition to site and size of open refuse deposits, open sewage and rainwater drainage systems, were geocoded and entered in a Geographic Information System (GIS) mapping database, as described previously [10], [11].

### Statistical analysis

Epidemiological and laboratory data were double-entered and validated using Epi-Info for Windows software (Centers for Disease Control and Prevention, Atlanta, GA). There were no missing values for any of the analyzed variables. We used proportions and medians with interquartile range to characterize signs of rodent infestation in case and control households.

Concordance between specific markers of current *Rattus novergicus* infestation (rat feces) and variables indicating present or prior rodent infestation (burrows and runs) was assessed by the kappa index statistic. We used Chi-square and Wilcoxon rank sum tests to compare socio-demographic and environmental characteristics of case and control households for categorical and continuous data, respectively. We used the same tests to compare households with and without domicile or peridomicile structural changes in the period between serological and environmental evaluations. These last analyses were performed for the groups of case and control households separately.

Environmental variables with p<0.1 in the bivariate analysis were included in a multivariate logistic regression analysis to identify independent predictors of risk for *Leptospira* transmission. As some of the exposure variables studied was correlated at two conceptual levels of risk, firstly, environmental variables that influence the rodent infestation level and secondly, variables that measure proxies for rodent infestation, we used a hierarchical approach [26], [27] to identify independent predictors of risk for *Leptospira* transmission. We built three multivariate logistic regression models using backward elimination. The first model included environmental variables previously described as associated with rodent infestation, such as refusal deposit, sewage and vegetation. The second model included rodent infestation variables, such as rodent runs, feces and burrows. The third and final model included variables retained (P<.05) from the first and second models. We used SAS software for Windows for these analyses [28].

To develop a practical prognostic risk score for each household, we weighted independent variables identified by logistic regression proportionally to their *β* regression coefficient values, as previously described [29]. The use of mutually adjusted weights per predictor is the standard methodology to develop a prognostic risk score [30]. A risk score was calculated for each household. We assessed the discriminative power of the score by using receiver operating characteristic (ROC) curves of sensitivity and specificity. Sensitivity and specificity measured the proportion of case and control households, respectively, which were correctly identified as such by the risk score. Score predictive ability (*C*-statistics) was classified as excellent (>0.80), good (0.70–0.79), fair (0.60–0.69), and poor (0.50–0.59).

## Results

As previously described, we enrolled 2,003 participants from 684 households in a community cohort study designed to measure risk factors and infection rates for leptospirosis [11]. Of these, 1,585 (79%), 1,324 (66%) and 1,394 (70%) participants completed the first, second and third year of follow-up, respectively. We identified 104 *Leptospira* infections in 97 participants residing in 80 households during the three year study period. We identified fifty-one infections (49%) in the first year, 26 (25%) in the second and 27 (26%) in the third year. In all but four cases (96%), *L. interrogans* serogroup Icterohaemorrhagiae was identified as the presumptive infectious serogroup based on agglutination titers. A majority of the case households (63, 79%) had a single participant with evidence of infection, while 17 (21%) case households had two participants. Seven participants had serologic evidence of two exposures. In addition to the 80 households defined as case-households we also included 115 households, out of a possible 186, meeting inclusion criteria as controls.

We performed environmental surveys in 189 (97%) of the 195 households (80 case and 109 control households). Six control households could not be inspected because no persons were present during at least three attempted visits. The final number of case and control households included in this study was 80 and 109, respectively. We observed that case households had a higher number of inhabitants and number of subjects enrolled in the cohort study than control subjects (4 [IQR: 4–6] vs. 4 [3]–[5], respectively, Table 1; and 4 [IQR: 2–5] vs. 3 [2]–[4], respectively, P<0.05). Other characteristics regarding environment and rodent signs, of case and control households with comparative bivariate values of p<0.1 (inclusion criterion for logistic regression analyses), are shown in Table 1.

**Table 1:** Rodent-related and environmental characteristics associated with *Leptospira* transmission among case and control households at the community study site, Salvador, Brazil.

Household characteristics	Case^a^ (*n* = 80)	Control^a^ (*n* = 109)	
	No. (%) or median (IQR)^b^		*P* c
**Demographics**			
No. of inhabitants	4 (4–6)	4 (3–5)	<0.05
Per capita income, US$/d	2.6 (1.5–3.8)	3.7 (2.4–5.5)	<0.01
**Premise type and details** d			
Distance from open sewer, m	23.2 (12.4–44.7)	21.4 (7.9–36.2)	0.54
Distance from open refuse deposit, m	74.4 (49.1–105.1)	65.1 (45.6–83.6)	0.19
Level above lowest point in valley, m	20.5 (10.5–30.2)	21.0 (13.6–34.9)	0.21
**Rodent access to food sources** d			
Exposed garbage^d^	45 (56)	44 (40)	<0.05
Other food & plants^d^	32 (40)	30 (27)	<0.1
Fruit trees	45 (56)	38 (35)	<0.01
Open stores of human food	19 (24)	13 (12)	<0.05
**Rodent access to water** d	33 (41)	20 (18)	<0.01
Standing water^d^	24 (30)	15 (13)	<0.05
**Harborage for rodents** d			
Lumber/clutter on ground^d^	58 (72)	66 (61)	<0.1
Other large rubbish^d^	51 (64)	51 (47)	<0.05
Dilapidated fences & walls^d^	24 (30)	21 (19)	<0.1
Plant-related^d^	70 (87)	84 (77)	<0.1
Presence of exposed earth	64 (80)	61 (56)	<0.01
Built on earthen slope^e^	54 (67)	44 (40)	<0.01
**Entry/access for rodent** d			
Structural deficiencies^d^	54 (67)	53 (49)	<0.01
Hole(s) in roof	50 (62)	49 (45)	<0.05
Un-plastered walls^f^	66 (82)	63 (57)	<0.01
**Rodent active signs** d			
Active signs^d^	63 (78)	46 (42)	<0.01
Rodent burrows	52 (65)	32 (29)	<0.01
Rodent runs	46 (57)	38 (35)	<0.01
*R. norvegicus* feces	53 (66)	25 (23)	<0.01

a Case and control households comprised of households in which cohort subject(s) with evidence of *Leptospira* infection resided and neighborhood households which were located >30 m of a case household and did not have a member with evidence of *Leptospira* infection during the study period, respectively.

b Median and inter-quartile range (IQR) values are shown for continuous variables.

c Values are not shown for non-significant associations.

d Categories and variable defined in the CDC form [22].

e Presence of exposed earth slope (>45°) within 10 m of the household.

f Walls composed of exposed bricks without external application of stucco or plastering.

We detected rodent infestation (presence of at least one rodent sign) in 63 (78%) of the case and 46 (42%) of control households. Rat burrows were frequent signs of rodent infestation (65% and 29% for case and control households, respectively). A total of 101 among the 189 households surveyed, had fecal droppings, 77% were from *R. norvegicus*, 10% from *M. musculus* and 4% from *R. rattus*; feces from a non-identified species were present at 9% households. Overall, the presence of *R. norvegicus* fecal droppings had good concordance with presence of any rodent burrow (kappa = 0.61) and a moderate concordance with any rodent run (kappa = 0.51).

Eighty (42%) households, 35 cases and 45 controls, had structural, sewage or trash modifications between the year of *Leptospira* infection and date of environmental survey. Case households with modifications were compared with case households lacking modifications. The same analysis was performed for control households. There were no significant differences among the study variables between groups (data not shown) and all 189 households were considered for further analyses.

In bivariate analyses, we identified 13 environmental variables which were associated (*P*<0.05) with case households (Table 1). A larger percentage of case households (*P*<0.01) showed signs of rodent infestation related to *R. norvegicus*. Additionally, the presence of a case of *Leptospira* infection in a household was associated with low socioeconomic status as per capita income and number of inhabitants in the house. Residents of a large number of case and control households were squatters, 91% and 86% respectively. We did not identify significant differences between case and control households with respect to the presence or number of dogs, cats or chickens (data not shown). Residents also reported ownership of other species of animals as ducks, small birds, rabbits, hamsters, monkeys and turtles, but the presence/number of these animals was not associated with differences in *Leptospira* infection among residents of case and control households.

The first multivariate logistic regression model, including variables related to househo*l*d environment, retained the following characteristics: rodent access to water, domicile built on a slope and un-plastered exterior wall surfaces. The second model, including rodent infestation variables, retained the presence of *R. norvegicus* fecal droppings and rodent burrows. The final model retained four variables: two household environmental variables and two rodent infestation factors (Table 2). *R. norvegicus* fecal droppings had the strongest association with case households in the final model followed by rodent burrows, rodent access to water and un-plastered exterior wall surface.

**Table 2:** Logistic regression analysis of rodent-related and environmental characteristics associated with *Leptospira* transmission and scoring system at household level.

Variables	OR (95% CI)^a^		*β* regression coefficient	Points^b^
	Unadjusted	Adjusted		
*R. norvegicus* feces	6.59 (3.46–12.55)	4.95 (2.13–11.47)	1.52	3
Rodent burrows	4.46 (2.41–8.28)	2.80 (1.06–7.36)	1.02	2
Access to water	3.12 (1.61–6.03)	2.79 (1.28–6.09)	0.99	2
Un-plastered walls^c^	3.44 (1.72–6.86)	2.71 (1.21–6.04)	0.90	2

a Odds ratios (OR) and 95% confidence intervals (CI) are shown for analyses. Logistic regression was performed to obtain estimates for odds ratios which were adjusted for covariates in the final model.

b Assignment of points to risk factors was based on a linear transformation of the corresponding *β* regression coefficient. The coefficient of each variable was divided by 0.90 (the lowest *β* value, corresponding to Un-plastered walls), multiplied by two, and rounded to the nearest integer.

c Walls composed of exposed bricks without external application of stucco or plastering.

**Figure 2:**
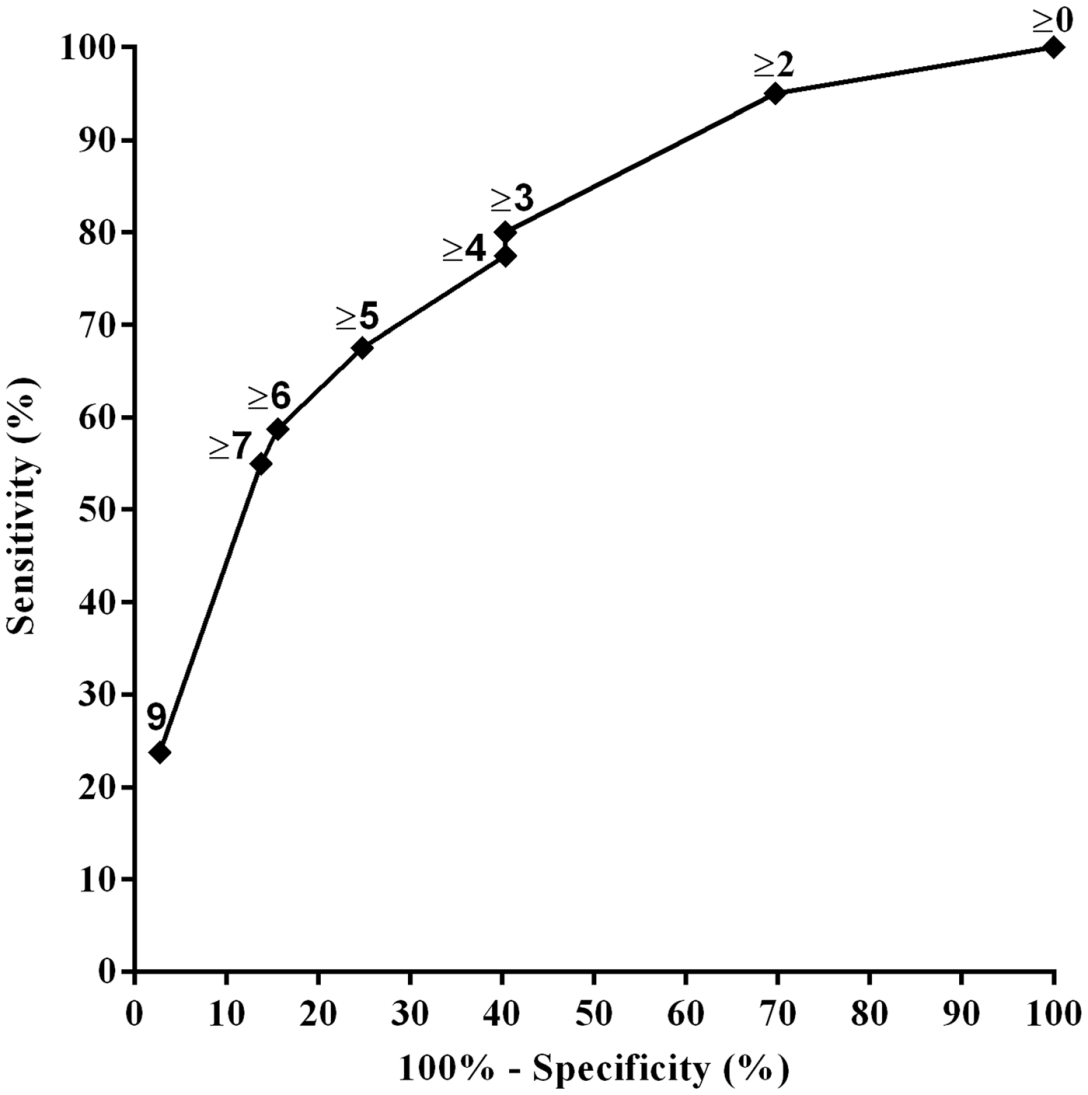
Receiver operating characteristic (ROC) curve for based logistic regression model score system. AUC (area under the curve) was 0.78 (95% CI 0.71–0.84).

To build a risk score, we assigned numerical scores to each of the four independent variables from the final model proportionate to the regression coefficient for each variable (Table 2). The sum of the number was used to classify each household into ten categories ranging from 0 to 9. None of the households received 1 or 8 points. Five percent of the case households and 30% of the control households had a score value of 0. Because score values were not normally distributed within case and control households, we used Wilcoxon rank-sum tests to compare the scores by case status. The median risk score for case households was 7, statistically different from the value of 2 for control households (*p*<0.001). Receiver operator curve (ROC) analysis yielded a very good to excellent *c* statistic of 0.78 (95 percent confidence interval: 0.71–0.84) (Figure 2). Table S2 presents the sensitivity, specificity and the estimated proportion of the case and cohort households for each score level.

## Discussion

High levels of rodent infestation and the predominance of *Rattus norvegicus* are frequent features within urban slum areas, in Brazil and around the world. Efforts to implement and improve rodent management interventions to reduce urban leptospirosis have been hampered by the lack of readily available information and epidemiologically-based markers that allow identification and monitoring of households at increased risk for infection. Our study demonstrates not only the large proportion of houses in a typical Brazilian slum at risk of acquiring *Leptospira* transmission, but also that the risk is significantly associated with four markers of rodent infestation and environmental factors (*R. norvegicus* feces, rodent burrows, access to water, un-plastered walls). The risk score system we developed, by weighting and combining values for each of these features, accurately classified households into risk groups for *Leptospira* infection with high precision. Of note, similar household-based markers of rodent infestation and infection risk have been described in association with Lassa fever [31] and hantavirus pulmonary syndrome [32]. We propose that targeting households at higher predicted risk for leptospirosis for augmented chemical, environmental and educational interventions would result in the greatest reduction of *Leptospira* transmission.

Our summary score value for high risk environments is easy to use and increases accuracy in identifying high risk households. The score performed well in discriminating case from control households with an accuracy of 0.78 and a sensitivity and specificity at a point value of 3 of 80% and 60%, respectively. These findings are encouraging and suggest that this tool could help inform more aggressive rat control to household locations with similar risk profiles without increasing the current workload of zoonotic control assessment teams.

Our scoring method could benefit other cities in Brazil and other countries where rodent control programs are the principal strategy to decrease leptospirosis incidence [20]. Rodent control programs are time-consuming and expensive, as they require large numbers of trained persons. In Salvador alone, the ZCC programs prioritize target areas with 20,000–60,000 households in locations where a high incidence of leptospiral disease (21.4 cases per 100,000 pop. in 2008 [unpublished data]) has been detected. Considering the coverage of the ZCC program in cities such as Salvador in Brazil and the leptospirosis incidence in these urban centers, it may be feasible to use changes of leptospiral disease incidence to evaluate the efficacy of enhanced targeted rat-control strategies which include the proposed score.

Although our study focused on a single area within Salvador, it would be useful to assess the utility of our measure to predict risk of leptospiral infection in other slum areas. However, this will require some tailoring of the survey instrument to some other locations, as every slum has unique characteristics and significant socioeconomic and environmental heterogeneities [33] within the broad definition for slum settlement as proposed by the United Nations [34]. Additionally, information regarding the incidence of leptospiral infection as determined by annual serosurvey is costly and labor-intensive, consequently such studies are a rarity. In the few reports where geocoded data for leptospirosis are available [35], spatial information has been restricted to outcomes of hospitalized cases of severe leptospirosis. The absence of other prospective studies to evaluate *Leptospira* infection prevented us from performing an external validation to evaluate the accuracy of our score in other settings. However, even with these limitations it may be possible to test the external validity of our survey methods and subsequent risk-scoring within a large area of Salvador and within other Brazilian cities where up to 33% of the urban population has equal or greater levels of poverty as found in our study community [23].

We identified two environmental risk factors, access to water and un-plastered walls, which were associated with an increased risk of *Leptospira* infection. Our finding that the presence of standing water, including sewer water, increases the risk of acquiring *Leptospira* infection, builds on our group's prior findings that household proximity to open sewers is associated with both *Leptospira* infection and severe disease in humans [5], [10], [11]. This may reflect the need of rats to a ready access of water as suggested by previous studies [36]–[38], and the capacity of open sewers to serve as environmental features contributing to the risk of leptospiral transmission to humans [9]. Infrastructural deficiencies, such as the presence of un-plastered walls in the home, were significantly associated with case households, and are a characteristic presumed to increase the probability of rat ingress into residences. However, *Rattus norvergicus*, which was the predominant rodent in the study area, typically resides outdoors and consequently it is more probable that un-plastered walls serve as a proxy for socioeconomic status than a proxy of rodent infestation. In conjunction, these specific household environmental and rodent infestation characteristics showed to be objective markers of *Leptospira* transmission.

This study provides further evidence of the importance of rats in urban leptospirosis transmission. The high household infestation rate, elevated *Leptospira* prevalence [15] and long term carriage [39] making *R. norvegicus* a major reservoir host for leptospires. We did not evaluate *Leptospira* carriage in domestic animals such as dogs. It is possible that dogs, which are often found in poor urban communities and may be infected with serovar Copenhageni [40], could contribute to the transmission cycle. However we think this is unlikely in our study area because this and other studies did not identify an epidemiological link between dogs and human *Leptospira* infection or leptospirosis [5], [10], [11].

We successfully identified environmental features associated with higher infection risk, but our study was limited by the time lag between occurrence of *Leptospira* infection and assessment of housing rodent survey. However, we showed that the four major causes of temporal modification in a household or environs (i.e. rebuilding or expansion; closing or creation of a nearby open sewer and removal or creation of refuse deposits since the year of *Leptospira* infection) did not influence environmental or infestation measures among case or control households. We did not evaluate the characteristics of the places where participants with *Leptospira* infection worked. However previous studies have found strong associations between risk of leptospiral transmission and environmental conditions around the case household, irrespective of work location [5], [10], [11], so we believe our findings are plausible and consistent.

We used serologically confirmed cases of subclinical *Leptospira* infection to define case households. Notwithstanding, it is likely that mild or subclinical *Leptospira* infection and clinical disease share the same environmental risk exposures. A previous study showed that members of households living with an index case of clinical leptospirosis were more likely to have serologic evidence for a prior infection than members of other households in the same communities [41]. Additionally, environmental deficiencies such as presence of open sewer near of the household and sighting rats in the peridomiciliary environment were independent risk factors for both severe leptospirosis [5] and *Leptospira* infection [10], [11].

In conclusion, we developed a risk score based on four variables related to objective signs of rodent infestation and environmental features that predict risk of *Leptospira* infection among persons living in households located within urban slums of Salvador. These findings have the potential to better inform policymakers and rodent management programs by identifying high-risk households and areas for frequent interventions and reducing effort directed at lower risk households and neighborhoods.

## Supporting Information

Table S1 English and Portuguese description of variables included in the modified exterior inspection form, adapted from the CDC manual.
(DOCX)

Table S2 Score system sensitivity, specificity and estimated proportion of the case and control households treated at each scoring category.
(DOCX)

Checklist S1 STROBE checklist.
(DOC)
