# Supplementary material for: Influence of Household Rat Infestation on Leptospira Transmission in the Urban Slum Environment
Source: PLoS Negl Trop Dis. 2014 Dec 4;8(12):e3338. doi: 10.1371/journal.pntd.0003338 (PMC4256176; doi:10.1371/journal.pntd.0003338)
Supplement: Table S1 — English and Portuguese description of variables included in the modified exterior inspection form, adapted from the CDC manual. (DOCX) [file pntd.0003338.s001.docx]

**Supplemental table 1.** English and Portuguese description of variables included in the modified exterior inspection form, adapted from the CDC manual.

| **Variables** | **CDC Survey form No.** ^a^ | **Comments** |
| --- | --- | --- |
| **Demographics** |  |  |
| Number of inhabitants | - | New |
| Proportion of males | - | New |
| Per capita income, US$/d | - | New |
| Squatter household | - | New |
| **Premise type and details** |  |  |
| Residential use only | 1 | Included |
| Commercial & residential | 2 | Included |
| Commercial | 3 | Not included |
| Vacant lot | 4 | Modified: Borders on a vacant lot |
| Food commercial establishment | 5 | Not included |
| Vacant | 6 | Not included |
| No. of dwelling units | 7 | Not included |
| Sewer on premise | 8 | Modified: Open sewer <10m |
| Distance from open sewer, m | - | New |
| Distance from open refuse deposit, m | - | New |
| Level above lowest point in valley, m | - | New |
| Borders an abandoned house | - | New |
| **Access to food sources** |  |  |
| Unapproved refuse storage | 9 | Not included |
| Exposed garbage | 10 | Included |
| Animal food | 11 | Included |
| Other food & plants | 12 | Included |
| Fruit trees | - | New |
| Open stores of human food | - | New |
| **Rodent access to water** |  |  |
| Standing water | 13 | Included |
| Condensate | 14 | Not included |
| Leaks | 15 | Included |
| **Harborage for rodents** |  |  |
| Abandoned vehicles | 16 | Included |
| Abandoned appliances | 17 | Included |
| Lumber/clutter on ground | 18 | Included |
| Other large rubbish | 19 | Included |
| Outbuildings /privies | 20 | Included |
| Dilapidated fences & walls | 21 | Included |
| Plant related | 22 | Included |
| Bushes or shrubbery | - | New |
| Ornamental plants | - | New |
| Presence of exposed earth | - | New |
| Built on earthen slope^b^ | - | New |
| **Entry/Access of rodents** |  |  |
| Structural deficiencies | 23 | Included |
| Pipe/wiring gaps | 24 | Not included |
| Hole(s) in roof | - | New |
| Hole(s) in wall | - | New |
| Hole(s) in floor | - | New |
| Un-plastered walls^c^ | - | New |
| **Signs of rodent infestation** |  |  |
| Active signs | 25 | Included |
| Rodent burrows | - | New |
| Rodent runs | - | New |
| *R. norvegicus* feces^d^ | - | New |
| *R. rattus* feces^e^ | - | New |
| *M. musculus* feces^f^ | - | New |
| **Number of domestic animals** |  | New |
| Dogs | - | New |
| Cats | - | New |
| Chickens | - | New |
| Others |  | New |

^a^Variable number in the CDC form [[22](#_ENREF_22)].

^b^Presence of uncovered terrain slope, with angle >45º, localized to a ≤ 10m of the household.

^c^Household walls without stucco application in the exterior surface and where is possible to observe the wall bricks.

^d^Capsule shaped and about 20mm long.

^e^Ellipsoid shaped and about 12mm long.

^f^Spindle shaped and about 6mm long.

| **Variáveis** | **Questionário ambiental CDC No.** ^a^ | **Comentários** |
| --- | --- | --- |
| **Demográficas** |  |  |
| Número de moradores | - | Nova |
| Proporção de homens | - | Nova |
| Renda *per capita*, US$/d | - | Nova |
| Invasão | - | Nova |
| **Características do domicílio** |  |  |
| Residência | 1 | Incluída |
| Comercial e residencial | 2 | Incluída |
| Comercial | 3 | Não Incluída |
| Local baldio | 4 | Modificada: Limita com casa abandonada |
| Comercio de alimentos | 5 | Não Incluída |
| Local vacante | 6 | Não Incluída |
| No. unidades habitacionais | 7 | Não Incluída |
| Esgoto no local | 8 | Modificada: Distancia a esgoto aberto, m |
| Distancia a esgoto aberto, m | - | Nova |
| Distancia a resíduos expostos, m | - | Nova |
| Nível acima do ponto mais baixo do vale, m | - | Nova |
| Limita com casa abandonada | - | Nova |
| **Aceso a fontes de alimento** |  |  |
| Armazenamento de lixo inadequado | 9 | Não Incluída |
| Resíduos expostos | 10 | Incluída |
| Alimento para animais | 11 | Incluída |
| Outros alimentos e plantas | 12 | Incluída |
| Árvores frutíferas | - | Nova |
| Armazenamento inadequado de alimento para humanos | - | Nova |
| **Acesso a água** |  |  |
| Água empoçada | 13 | Incluída |
| Condensação | 14 | Não Incluída |
| Vazamentos | 15 | Incluída |
| **Refúgio para roedores** |  |  |
| Carros abandonados | 16 | Incluída |
| Artefactos abandonados | 17 | Incluída |
| Serralha ou entulho | 18 | Incluída |
| Resíduos maiores | 19 | Incluída |
| Galpões/banheiros externos | 20 | Incluída |
| Cercas e muros | 21 | Incluída |
| Plantas e derivados | 22 | Incluída |
| Arbustos | - | Nova |
| Plantas ornamentais | - | Nova |
| Peridomicílio com chão de terra | - | Nova |
| Ladeira de terra^b^ | - | Nova |
| **Acesso ao domicílio** |  |  |
| Deficiências estruturais | 23 | Incluída |
| Buracos (tubulação/eletricidade) | 24 | Não Incluída |
| Buraco(s) no teto | - | Nova |
| Buraco(s) no muro | - | Nova |
| Buraco(s) no chão | - | Nova |
| Parede não rebocada^c^ | - | Nova |
| **Sinais de infestação por roedores** |  |  |
| Sinais ativas | 25 | Incluída |
| Tocas de roedores | - | Nova |
| Trilhas de roedores | - | Nova |
| Fezes de *R. norvegicus*^d^ | - | Nova |
| Fezes de *R. rattus*^e^ | - | Nova |
| Fezes de *M. musculus*^f^ | - | Nova |
| **Número de animais domésticos** |  | Nova |
| Cães | - | Nova |
| Gatos | - | Nova |
| Galinhas | - | Nova |
| Outros |  | Nova |

^a^Número da variavel no questionario ambiental do CDC [[22](#_ENREF_22)].

^b^Presenca de ladeira de terra, com angulo >45º, localizada a ≤ 10m do domicilio.

^c^Domicilio com paredes nao rebocadas na superficie exterior e onde é possivel observer os tijolos do muro.

^d^Forma capsular de aproximadamente 20mm de cumprimento.

^e^Forma fusiformes de aproximadamente 12mm de cumprimento.

^f^ Forma fusiforme de aproximadamente 6mm de cumprimento.
