# Supplementary material for: Influence of Household Rat Infestation on Leptospira Transmission in the Urban Slum Environment
Source: PLoS Negl Trop Dis. 2014 Dec 4;8(12):e3338. doi: 10.1371/journal.pntd.0003338 (PMC4256176; doi:10.1371/journal.pntd.0003338)
Supplement: Table S2 — Score system sensitivity, specificity and estimated proportion of the case and control households treated at each scoring category. (DOCX) [file pntd.0003338.s002.docx]

**Supplemental table 2.** Score system sensitivity, specificity and estimated proportion of the case and control households treated at each scoring category.

| **Risk categories**  **(Score points)** | **Cumulative sensitivity (%)** | **Cumulative specificity (%)** | **Estimated proportion of the case and control households** |
| --- | --- | --- | --- |
| **≥2** | 95.0 | 30.3 | 72.8 |
| **≥3** | 80.0 | 59.7 | 45.2 |
| **≥4** | 77.5 | 59.7 | 44.9 |
| **≥5** | 67.5 | 75.2 | 30.0 |
| **≥6** | 58.7 | 84.4 | 20.9 |
| **≥7** | 55.0 | 86.2 | 18.8 |
| **9** | 23.7 | 97.2 | 5.3 |
